# When the Tube Won’t Go: Strategies for Difficult Nasogastric Intubation

**DOI:** 10.7759/cureus.96725

**Published:** 2025-11-12

**Authors:** Hashim Vakil, Anoir Lagzouli, Emily Lowe

**Affiliations:** 1 Orthopaedic Surgery, Southampton General Hospital NHS Foundation Trust, Southampton, GBR; 2 Trauma and Orthopaedics, Southampton General Hospital NHS Foundation Trust, Southampton, GBR; 3 Otolaryngology-Head and Neck Surgery, Southampton General Hospital NHS Foundation Trust, Southampton, GBR

**Keywords:** difficult insertion, feeding method, fne, general otolaryngology, nasogastric tube (ngt)

## Abstract

Nasogastric tube (NGT) insertion is a common yet sometimes technically challenging clinical procedure, essential in patients unable to swallow safely. Whilst generally straightforward, anatomical variations, prior surgery, trauma or neurological impairment may complicate the procedure. In such cases, otolaryngologists are often called upon to perform NGT placement under direct visualisation using flexible nasendoscopy (FNE).

This narrative literature review examines adjunctive techniques and strategies available to enhance the success of difficult NGT insertions, particularly those performed under FNE guidance in awake patients.

A comprehensive search was conducted using PubMed and Google Scholar, from 2005 to 2025, employing the MeSH term ‘nasogastric tube’ and other terms such as ‘difficult insertion’ and ‘insertion technique’. Studies were included if they involved adult awake patients and described alternative or adjunctive methods to facilitate NGT placement, with studies being excluded from the literature review if they involved anaesthetised patients. Data were manually extracted by multiple authors on study design, technique and outcomes.

Eleven relevant publications were identified. Techniques that increased success rates included prechilling or partially freezing the NGT to increase rigidity, the sniffing position, orientation, contralateral rotation and twisting (SORT) manoeuvre and FNE-assisted methods using surgical knots or dissolvable gel caps to guide the tube. Whilst these adjuncts demonstrated procedural efficiency and improved operator control, statistical improvement in success rates was not consistently shown. Nonetheless, patient comfort and procedural independence were notably improved with certain FNE-guided techniques.

Difficult nasogastric intubation remains a significant clinical challenge. Evidence suggests that combining physical modifications to the tube with direct visualisation via FNE can enhance safety and efficiency, particularly in anatomically complex or high-risk patients. Although advanced adjuncts such as gel caps and surgical knots have not demonstrated definitive superiority, they offer practical advantages in selected cases. Further randomised controlled trials (RCTs) are warranted to evaluate their efficacy in awake patients and to establish standardised protocols for difficult NGT insertion.

## Introduction and background

Nasogastric tubes (NGTs) serve as an essential and safe alternative method to providing essential nutrition support to patients who have undergone major abdominal or head and neck surgery, those who have dietary disorders requiring extra nutritional support and those patients who have suffered a stroke or other neurological pathology rendering them unable to swallow without aspirating into the lungs. Although a relatively simple procedure in a compliant patient, there are times when there is great difficulty in getting an NGT into the oesophagus. Following unsuccessful attempts, an otolaryngologist may be called upon to perform the procedure under direct visualisation through flexible nasendoscopy (FNE).

This narrative literature review aims to explore adjunctive methods and techniques available to otolaryngologists, which may be used alongside FNE to increase rates of successful NGT insertions amongst patients in whom NGT insertion is difficult.

Anatomy and standard procedure

**Figure 1:**
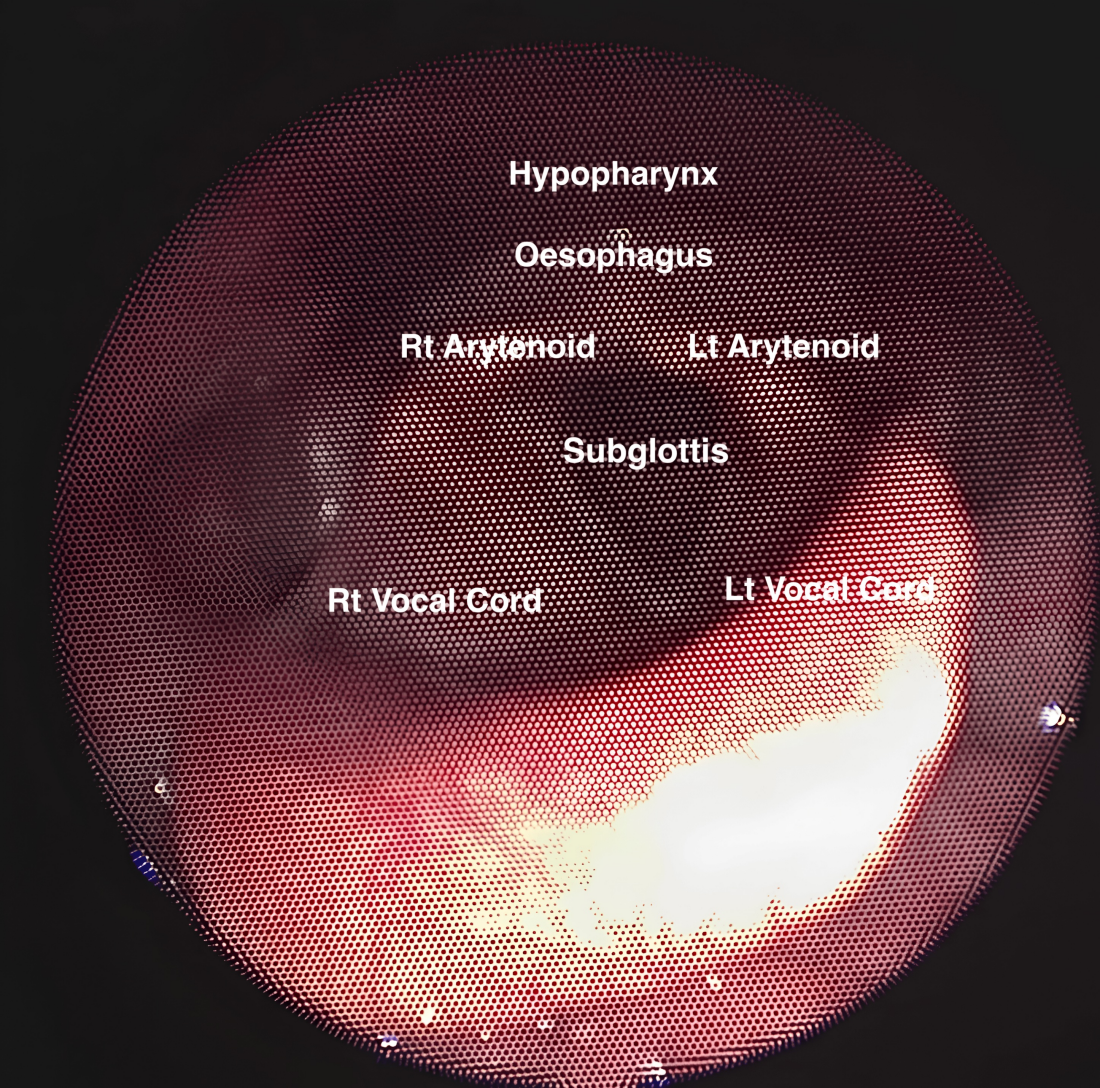
Labelled and live image as viewed through laryngoscopy or flexible nasendoscopy (FNE). Basic view of the vocal cords and subglottic area, asking the patient to stick their tongue out. Consent from the patient to submit images has been obtained prior to submission to the journal.

**Figure 2:**
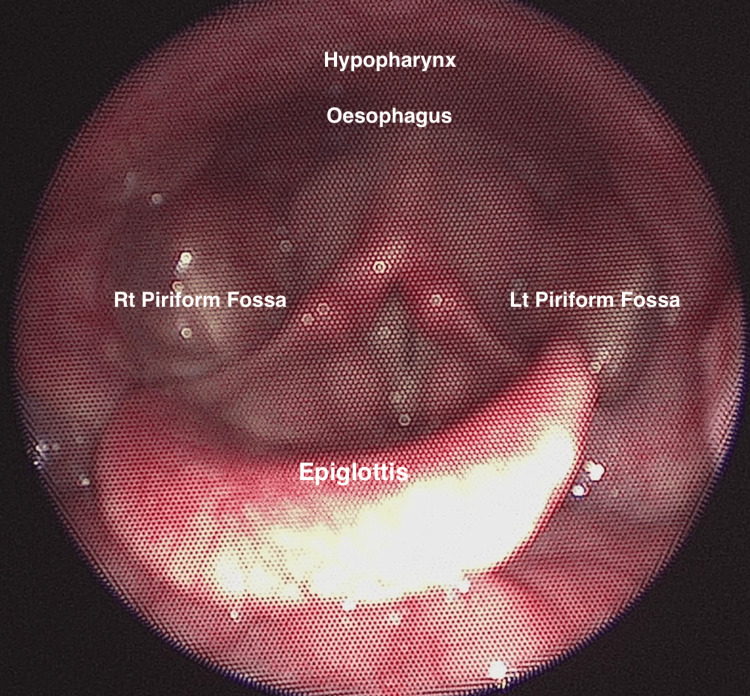
Labelled and live image as viewed through laryngoscopy or flexible nasendoscopy (FNE). Basic view of the piriform fossa, asking the patient to close their mouth and puff their cheeks with air. Consent from the patient to submit images has been obtained prior to submission to the journal.

When visualising the anatomy of the oesophagus in relation to the larynx during a flexible nasendoscopy, we focus on several key structures in the upper airway. The larynx is situated at the level of the C3-C6 vertebrae in adults and lies directly in front of the cervical spine. The main components of the larynx include the epiglottis, the vocal cords and the arytenoid cartilages (Figure 1). The pharynx is also examined, including the nasopharynx, oropharynx and hypopharynx. The upper oesophagus is located posterior to the larynx in the post-cricoid space, flanked on either side by the piriform fossae sinuses (Figure 2).

Indications

The nasogastric tube may be considered in several clinical scenarios. Most simply, it is used to deliver contents to the stomach and to remove contents from the stomach, such as seen in the case of bowel obstructions, as highlighted by the Cleveland Clinic [1].

Commonly, it is used in cases of dysphagia and sequelae of ischaemic or haemorrhagic intracerebral event or due to a malignancy such as a tongue base tumour, making swallowing almost impossible for those patients. It has also been used for cases of severe malnutrition in such scenarios of refeeding syndrome and in patients who have inflammatory bowel disease, making optimal nutritional supplementation rather difficult to achieve.

Nutrition is important in optimising outcomes post-operatively, making the NGT an integral part of recovery. As detailed by Talwar et al., ‘early nutrition reduces morbidity’ and the nutritional status of the patient will determine further treatments available to the patient [2]. Poor nutritional status may lead to inadequate wound healing, slow recovery post-operatively and muscle atrophy, ultimately leading to more unfavourable outcomes secondary to increased frailty due to malnutrition [3].

An important indication of the NGT in patients with dysphagia is for the administration of their medication [4]. Patients who have presented following strokes may require antiepileptic medications to ensure adequate seizure control. Similarly, a patient with Parkinson’s disease may struggle with the self-administration of medication orally and require assistance through the NGT. In the absence of intravenous alternatives, as demonstrated in medications such as lamotrigine, an NGT may be an adequate adjunct in ensuring that healthcare needs are met. Careful consideration should be made when passing medication through the NGT, as particular medications should not be crushed prior to administration, making it not suitable to pass through the NGT; these include enteric-coated medications such as aspirin or certain sustained-release medications such as carbamazepine [5]. If uncertain, all necessary medication should be discussed with a pharmacist to ensure patient safety.

In the case of a patient with skull-base fractures, the FNE may be extremely useful to ensure the safe passage of the NGT through the nasal cavity. The biggest risk of inserting the NGT in a skull-base fracture is inadvertently inserting the NGT into the cranial cavity through the fracture site. A couple of case reports have been published highlighting a case of intracranial NGT insertions following septoplasty [6], as well as intracranial insertion in a patient with severe craniofacial trauma [7]. This highlights the importance of considering FNE-guided NGT insertions in cases involving facial fractures or operative procedures involving the facial bones. In such cases, had an NGT been inserted using an FNE under direct visualisation, it may have been possible for complications and subsequently further harm to the patient to have been ultimately avoided.

Following the safe passage of the NGT through the nasal cavity into the nasopharynx and into the laryngopharynx, the FNE alone simply provides visualisation and confirms the position of the NGT. It allows immediate recognition of intra-tracheal insertion or correct oesophageal insertion.

It has been a common practice in general surgery that NGT would be used post-operatively to hasten bowel recovery and bowel function, as well as prevent the risk of aspiration and aspiration pneumonia by decompressing the stomach and the bowel. This function of the NGT is commonly utilised in bowel obstruction to help decompress the bowel. The usage of the NGT has been looked in multiple published studies, asking the sensible question of whether we really need to use the NGT to decompress the bowel. A Cochrane review done by Verma and Nelson, carried out in 2007, suggested that the use of the NGT post-operatively provided a statistically significant earlier return of bowel function and decrease in pulmonary complications [8]. Despite its benefits, there were noticeable disadvantages highlighted by the study, such as patient discomfort and increased episodes of vomiting in patients with an NGT inserted. As mentioned in the authors’ conclusions, the careful consideration of the NGT in these patients is essential for positive patient outcomes.

Practice in the management of small bowel obstruction (SBO) has changed over the years as further focus on the NGT has been studied. A paper published in 2022 by Ball looked at Dr Livingston and colleagues and their patient cohort, assessing the use of the NGT in an SBO [9]. It was ultimately published that those who have a moderate physiological reserve and a partial SBO could be managed without an NGT. A similar study was carried out by Fonseca et al., where 190 patients with SBO were managed conservatively, and 55 of them did not receive an NGT [10]. It was determined that the population that received the NGT did not present with emesis; however, the development of pneumonia and respiratory failure was significantly associated with the placement of an NGT. Equally, the usage of the NGT increased the length of stay within hospitals for patients occupying beds within the hospital for a longer period.

## Review

Methods

Data Searches and Search Strategies

A comprehensive literature search was conducted using the PubMed database to identify studies relevant to difficult NGT insertions. The search terms included ‘Nasogastric tube’ AND ‘Difficult insertion’, ‘Difficult Nasogastric tube insertion’ and ‘Insertion technique’. Additional synonymous terms such as ‘Enteral Tube’ and ‘Feeding Tube’ were also incorporated.

Inclusion and Exclusion Criteria

Articles were included if they were studies of adult patients and written in the English language. The review of the literature takes special interest in patients who are awake. This specifically excludes patients who are intubated or sedated in either the theatre or the intensive care unit. Papers involving patients who had specific pathologies or diagnoses that would make inserting a nasogastric tube difficult were also considered and included as part of the literature review. Despite low strength in evidence, abstracts that involved unique techniques utilised by practitioners were included and considered as part of the literature review. Posters and poster presentations were excluded from the study.

Data Extraction

All data extraction and review processes were performed manually by the primary reviewer and two other contributing authors without the use of automated tools or artificial intelligence. Data were extracted on the following variables of interest: publication year, study design, treatment and outcomes. Studies describing theoretical or alternative techniques for NGT insertion were included. We accept that abstracts hold less strength than randomised controlled studies; however, techniques and unique modalities presented by clinicians are worth highlighting in order to show alternative approaches that may be later scientifically studied.

Search of the Literature

The search term was as follows: ‘Nasogastric Tube’ OR ‘Feeding Tube’ OR ‘Enteral Tube’, which yielded an initial total of 45,297 publications on PubMed. The refinement of the search with the additional term ‘AND difficult insertion’ reduced the yield to 183 publications. To further narrow the selection to relevant awake patient populations, additional exclusion criteria were applied: ‘NOT Anaesthetised’ and ‘NOT Intubated’, resulting in 82 publications. Of these 82, there were no obvious repeated publications; duplicates were assessed manually by the authors. Through the initial screening process of reviewing publications by their titles and abstracts, a further 71 publications were eliminated from usage in this study, resulting in a remaining 11 publications. A comprehensive review was then carried out, excluding a further six publications from the study. These publications were excluded as they did not involve any techniques or descriptions of a technique used to help aid NGT insertion and did not meet the inclusion criteria. The remaining five papers were retrieved from the PubMed database search, Google Scholar, Medscape and ScienceDirect.

**Figure 3:**
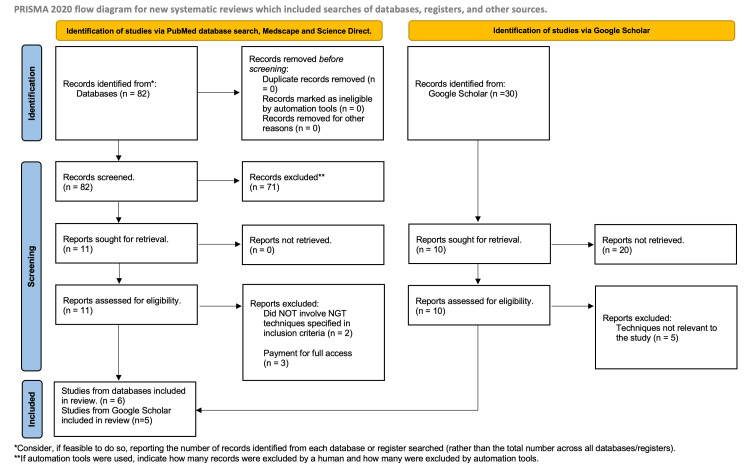
PRIMSA chart for literature review. PRISMA, Preferred Reporting Items for Systematic Reviews and Meta-Analyses; NGT, nasogastric tube

Additionally, articles and grey literature were sought out through Google Scholar. A total of 158 searches were identified when searching ‘Difficult NGT insertion techniques’ through the Google search engine; of these, there were 30 relevant articles identified. Of the 30, 10 were assessed for eligibility, with five articles being used in addition to the PubMed publications mentioned earlier. This brought the total number of publications and articles used in the review to 11 (Figure 3 and Table 1).

**Table 1:** Tabular literature summaries from the literature review. FNE, flexible nasendoscopy; SORT, sniffing position, orientation, contralateral rotation and twisting; ICU, intensive care unit

Authors	Origin	Purpose	Type of Source	Research Design	Summary Points
Flegar and Ball (2004) [11]	Association of Anaesthetists	Informative	Publication	Descriptive	Describing the technique of chilling and keeping nasogastric tube (NGT) in the refrigerator to help keep it rigid with ‘memory’ for its coiled shape
Mazlom et al. (2020) [12]	Journal of Caring Sciences	Comparative study	Publication	Randomised study	Comparing cold to normal-temperature nasogastric tube and how they affected the administration of the NGT
Der Kureghian et al. (2011) [13]	The Journal of Laryngology & Otology	Informative/descriptive	Publication	Descriptive	Introduction of a technique using silicone alginate dressing to knot and tie the FNE to an NGT
Ahmad et al. (2015) [14]	Annals of the Royal College of Surgeons of England	Description of a new technique	Letter	Descriptive	A letter highlighting a surgeon’s technique of inserting a difficult NGT using a specific surgical knot
Upile et al. (2011) [15]	Head & Neck Oncology	Comparative study	Publication	Prospective comparative study	Comparing the use of a gel cap to facilitate the NGT insertion to normal routine NGT insertions highlighting the gel cap to not be statistically more impactful in a successful NGT insertion than the original blind technique
Lin et al. (2006) [16]	Gastrointestinal Endoscopy	Consecutive prospective study	Publication	Consecutive prospective study	The use of guidewires to help insert an NGT into patients with abnormal anatomical pathologies (oesophageal cancer)
Chun et al. (2009) [17]	World Journal of Surgery	Comparative study	Publication	Randomised clinical trial	Comparing frozen to standard NGT placements in anaesthetised patients. Overall, highlighting that freezing the NGT increases the success rate of inserting an NGT in intubated patients
Mandal et al. (2014) [18]	Indian Journal of Anaesthesia	Comparative study	Publication	Randomised clinical trial	Compared four techniques in intubated patients to assess which technique was superior. Highlighting that the three techniques studied were all better than the original conventional method of inserting an NGT
Mandal et al. (2018) [19]	Indian Journal of Anaesthesia	Comparative study	Publication	Randomised clinical trial	Compared three techniques in intubated patients of inserting an NGT. Ultimately determining that, statistically, Sellick’s manoeuvre was the most effective
Najafi and Golzari (2016) [20]	Association of Anaesthetists	Informative/descriptive	Publication	Descriptive	Highlighting the SORT manoeuvre and how to implement it
Sanaie et al. (2020) [21]	Annals of Intensive Care	Comparative study	Publication	Prospective randomised clinical trial	A study looking at the SORT manoeuvre versus neck flexion and lateral pressure in ill patients admitted to the ICU

Literature review

When common ward-based attempts at inserting the NGT fail or certain skull-base fractures make the insertion of the NGT risky, it is common practice, institutionally, to request assistance from the otolaryngologists to insert an NGT with an FNE. The FNE, as mentioned, provides the direct visualisation of the nasal passage and the nasopharynx all the way to the larynx and the oesophagus.

Prior to reviewing the literature on FNE-guided insertions of NGTs, consideration needs to be given to techniques that may be performed on the ward by either the nursing staff or any available clinician with the experience to insert an NGT. Classically, if the NGT insertion initially fails, then it may be recommended, by an anaesthetist or an experienced nurse, to chill the NGT in the refrigerator. As mentioned by Flegar and Ball (2004), published in the Association of Anaesthetists, the cooled NGT keeps the tube rigid with ‘memory for its coiled position’ [11]. The principle had been taken from a procedure called a pulmonary artery catheter insertion, where it was felt that maintaining the coiled characteristic of the catheter encourages easy access to the heart, especially through the left subclavian. Similarly, the coiled NGT allows for an angled approach to the oesophagus, giving the clinician an angle to advance towards the oesophagus and pass the NGT into the oesophagus. A randomised clinical trial involving 100 patients was carried out in a toxicology emergency department to test the effectiveness of chilling the NGT in a refrigerator prior to insertion (n=50) in comparison to keeping the NGT at room temperature (n=50). According to Mazlom et al. (2020), when the NGT was left at room temperature, the success rate for first-attempt insertions of the NGT was 84.4% in comparison to chilling the NGT in the range of 2-8 degrees Celsius, which results in a 100% success rate in first-time NGT insertion in their patients [12].

If attempts to insert an NGT have failed, the utilisation of a flexible nasendoscopy (FNE) may be considered. The FNE allows for the direct visualisation of the larynx, as mentioned in the text earlier. Alone, it may provide usefulness in visualising where the NGT is pushing within the larynx, as well as its usefulness in determining anatomical variants that are making the insertion difficult. However, when attempting to overcome the difficult variant, it alone may not be sufficient; improvisation and innovation may be required by the clinician to ensure that the NGT is passed into the proximal oesophagus.

One method was described by Der Kureghian et al. (2011), where the FNE optimises the insertion by providing direct visualisation that you would not have in the blind techniques [13]. In this method, the NGT is anchored to the FNE using sodium alginate dressing ties and enters the larynx via the nasal cavity. Once the position has been confirmed and the NGT has passed into the proximal oesophagus, it is advanced, whilst the FNE is pulled away, causing tension. This gentle tension allows the loosening of the ties, which frees the FNE from the NGT. This, in theory, is a relatively safe method as the sodium alginate dressings are safe to ingest and have been used in previous instances to treat gastric ulcers. As mentioned in the literature, a significant amount of local anaesthetic will need to be utilised as you are passing two small lumen tubes through a patient’s nasal cavity. Without this, there is a possibility of making the patient uncomfortable and subsequently failing the procedure.

In a paper published by Ahmad et al. (2015), a method was proposed involving tying the FNE with three surgical ties, allowing for free movement and the guidance of the NGT using the FNE [14]. Once the FNE guides the NGT into the oesophagus, forceps are used to hold the FNE in place, whilst the NGT is removed by undoing the surgical knots. The surgical knots remain with the NGT until the NGT is officially removed. However, this idea has not been clinically tested, with no evidence of statistical advantages to performing this procedure. Based on the technique described, a theoretical infection-control risk may be identified.

A novel technique described and studied by Upile et al. (2011) describes using the gel cap to insert a laryngectomy speech valve as a conduit [15]. The gel cap keeps the FNE tip and the NGT attached, allowing for the movement of the NGT to be facilitated by the FNE. The benefits of this method over the surgical knot procedure described above are that the gel capsule is dissolved by the gastric contents of the stomach, meaning that there is no concern with foreign bodies or objects remaining in the patient’s body once correctly inserted. Both procedures highlight the clear advantage of being able to perform this procedure independently. Despite the theoretical advantages of carrying out the NGT insertion this way, the study carried out by Upile et al. ultimately revealed that there was no statistical advantage to using the gel cap to aid in successful NGT placement on an awake patient [15]. However, the study did reveal several notable advantages: the procedure was completed more quickly with the gel cap and required significantly less assistance. This makes it an ideal option for a lone physician performing the procedure. Additionally, patients reported greater comfort when the gel cap was used, with a mean discomfort score difference of 6.63, indicating that the technique was significantly more comfortable than the one performed without the gel cap.

A technique described by Lin et al. (2006) talks about the application of a nasogastric feeding tube using an ultrathin trans-nasal endoscope [16]. This paper reported its effectiveness in those with oesophageal cancer, making the anatomy rather difficult for the tube to traverse. The tube in question is an ultrathin 6 mm-diameter tube that may be passed through the nasal cavity, very similar to the FNE. In this study, the success rate was 99% with a single failure due to the oesophageal cancer causing a total obstruction, preventing the guidewire from passing. Although this technique does not require the use of an FNE, it does introduce the idea of utilising guidewires to ensure entry into the proximal oesophagus. It may be utilised in a similar way to the guidewire during intubation to ensure the safe passage of the endotracheal tube. In theory, this would minimise the risk of further injury to local structures from blunt trauma.

Discussion

Nasogastric tube (NGT) placement in awake patients is a frequently performed procedure that is generally straightforward in routine clinical practice. However, in a subset of patients, the procedure can prove challenging, often requiring multiple attempts and specialist input. In exceptional cases, such as base of tongue cancers and other macroscopic laryngeal cancers, as well as anatomically abnormal pathology, such as in post-radiotherapy fibrosis, direct visualisation may be sought out to ensure the safe and accurate insertion of a nasogastric tube.

Techniques described above may be useful to the physician attempting NGT insertion under direct FNE visualisation. As discussed, under direct visualisation without the modality to manipulate the NGT, it can be a rather challenging situation for both physician and patient. Consideration in using the gel capsule or surgical knots to secure the NGT to the FNE provides the physician with theoretical control over the movement of the NGT. This may provide minimal trauma to the patient and a sense of stability to the physician when inserting the NGT. Despite its theoretical advantages, there is no statistical evidence that the usage of an appliance increases the rate of a successful NGT insertion.

In a randomised clinical trial, carried out in 2009, conducted on intubated patients by the department of anaesthesiology and pain medicine at Bundang Medical Center, which reviewed a technique of filling the NGT with distilled water and freezing it, the success rate of passing the NGT correctly was higher than without partially freezing the NGT [17]. This was statistically significant with an increased success rate of 30% when freezing the NGT with distilled water (control/frozen=58%/88%; p=0.001). A key aspect of this study is that there was no difference in the incidence of epistaxis, a relatively common complication of inserting an NGT, which otolaryngologists may be expected to also manage.

Whilst techniques developed in anaesthetised patients, such as the reverse Sellick’s manoeuvre, show high success rates, their applicability in awake patients remains limited [18,19]. The physiological differences and tolerability thresholds in conscious individuals may preclude the routine use of these manoeuvres outside of controlled environments such as operating theatres or intensive care units.

Another technique that has been explored, commonly applied in anaesthetised patients, is the SORT manoeuvre, with SORT serving as a mnemonic for sniffing position, NGT orientation, contralateral rotation and twisting movement [20]. This approach has been described as feasible in both intubated patients and those who are critically ill and unable to swallow, making nasogastric tube (NGT) insertion particularly challenging. A clinical trial, published by Sanaie et al. (2020), involving 396 critically ill patients aged over 18, was conducted across two tertiary referral centres [21]. The participants in this study underwent either the SORT manoeuvre or the neck flexion with lateral pressure (NLFP) method, the latter being the standard approach at these institutions.

The trial revealed that the rate of failed attempts was significantly higher in the NLFP group compared to the SORT group (p<0.001), with the number of failed attempts showing the same correlation (P=0.046). The ease of insertion was reportedly greater in patients who underwent the NLFP technique in comparison to those who received the SORT manoeuvre. Nevertheless, the SORT technique was associated with a significantly higher rate of first-attempt success for NGT placement.

When employing the SORT manoeuvre in critically ill patients, consideration must be given to the flexibility and rigidity of the patient’s neck, as well as their ability to comply with the manoeuvre. It may be particularly difficult in patients with certain pathologies that limit physical compliance. This study underscores the importance of minor adjustments in both patient positioning and NGT orientation during insertion, which may enhance the likelihood of successful placement.

In summary, a combination of simple preparatory steps, anatomical understanding and the selective use of visual guidance can significantly improve outcomes in difficult NGT placements. Whilst evidence for certain advanced techniques remains preliminary, their pragmatic value suggests that they may be worthy of broader adoption and further investigation.

## Conclusions

Nasogastric tube insertion remains a vital clinical procedure in the nutritional and surgical management of patients who are unable to safely swallow or require gastrointestinal decompression. Whilst traditionally performed blindly at the bedside, the literature demonstrates that several adjunctive techniques can significantly improve success rates, particularly in difficult or anatomically complex cases. Techniques such as chilling the NGT or filling it with frozen distilled water offer low-resource, effective solutions for frontline clinicians. In cases where blind attempts fail or carry significant risk, such as in patients with skull-base fractures or distorted anatomy, flexible nasendoscopy (FNE)-guided insertion offers a safe and direct visual method for accurate placement.

Though the adjunctive use of tools and single-operator settings have not demonstrated statistically superior outcomes in terms of success rates, they have shown procedural and patient comfort benefits that merit consideration, particularly in settings where lone operators are common. Moreover, whilst high success rates are reported in anaesthetised patients using manoeuvres such as the reverse Sellick’s manoeuvre, the application of these findings to awake patients remains limited. Ultimately, the literature underscores that whilst no single technique guarantees success, a tailored approach offers the best chance for safe and efficient nasogastric tube placement. Further research into combining these methods in a controlled manner, especially in awake patients, would be beneficial to standardising practice and improving patient outcomes.
